# Sensing the spectrum: diverse stimuli for the NLRP3 inflammasome

**DOI:** 10.1038/s42003-026-10207-x

**Published:** 2026-05-20

**Authors:** Nanyang Xiao, Zexuan Lu, Jueqi Chen

**Affiliations:** 1 Department of Microbiology, University of Chicago, Chicago, IL USA; 2 Howard Taylor Ricketts Laboratory, University of Chicago, Lemont, IL USA

**Keywords:** Bacteria, Inflammasome, Virology

## Abstract

The NLRP3 inflammasome is a central inflammatory signaling pathway in host defense, cancer, metabolic disorders, and neurodegenerative diseases. Unlike other pattern-recognition receptors, NLRP3 senses perturbations in organelle integrity and ion homeostasis, enabling its activation by a remarkably broad spectrum of pathogen-derived and cellular stress signals. In this review, we summarize recent advances that have expanded our understanding of the diverse classes of NLRP3 stimuli and highlight breakthroughs in elucidating the molecular mechanisms underlying both infection-driven and sterile inflammation. Finally, we discuss unresolved questions surrounding the physiological relevance of NLRP3 stimuli and the nature of uncharacterized stimuli in sterile inflammatory diseases, emphasizing how resolving these gaps may inform targeted therapeutic development.

## Abstract (web summary)

A review summarizes recent progress that has broadened insight into the wide range of NLRP3 inflammasome stimuli and highlights key advances in uncovering the molecular processes that drive infection associated and sterile inflammatory responses.

## Introduction

**Fig. 1:**
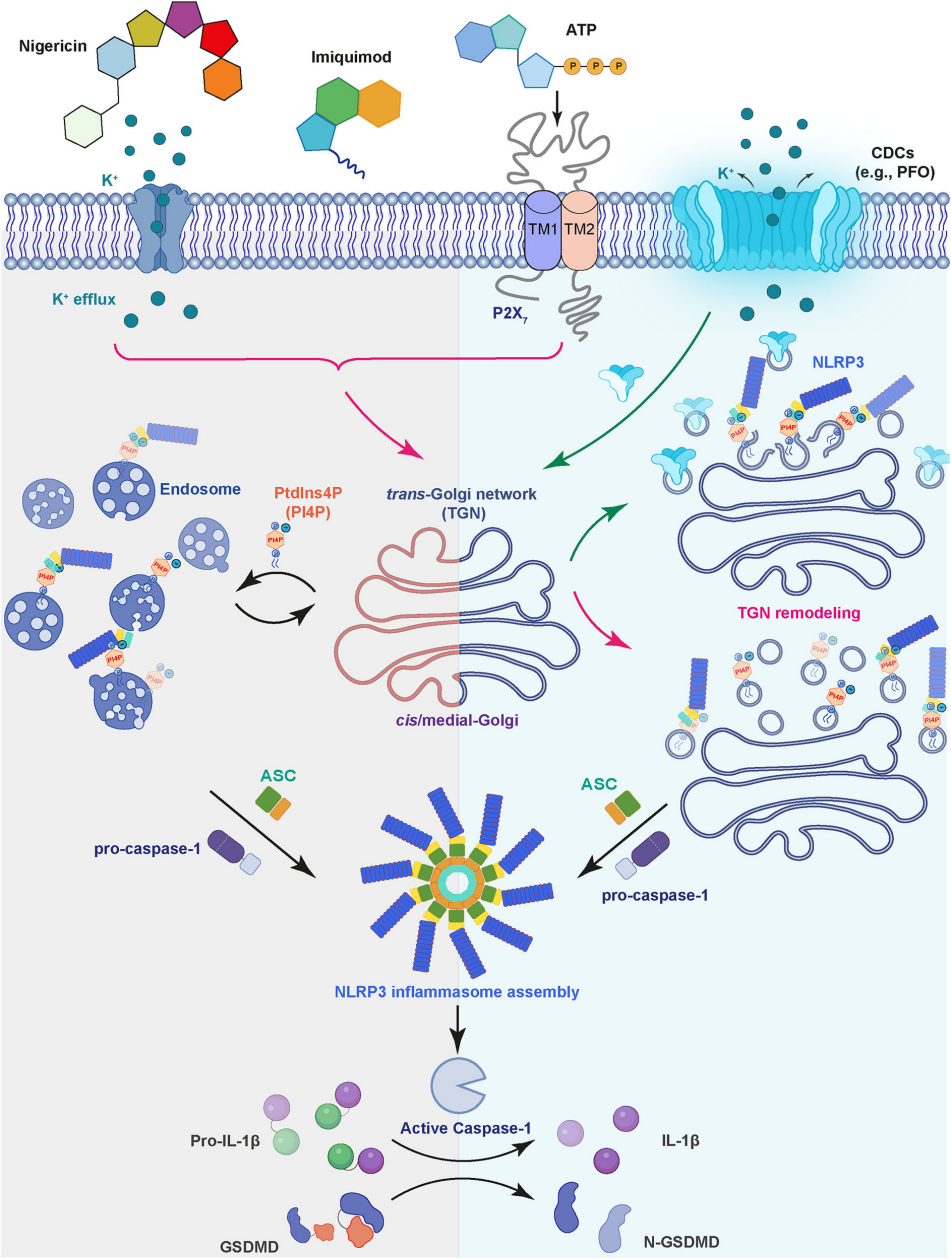
Model of TGN and endosomal remodeling by NLRP3 stimuli. Nigericin, ATP, and bacteria-derived toxins cholesterol-dependent cytolysins (CDCs) induce two parallel events, K^+^ efflux and TGN remodeling. In the case of nigericin and ATP (through the purinergic receptor P2X_7_) stimulation (red arrow), TGN is dispersed into multiple giant vesicles. The vesicles then recruit NLRP3 through an ionic bonding with the TGN-localized phospholipid PtdIns4P, a process enabled by K^+^ efflux. In the case of CDC (e.g., perfringolysin O (PFO)) stimulation (green arrow), CDC toxins are internalized inside host cells and traffic to the TGN, where they peel away PtdIns4P-negative membrane, thus exposing the relaxed PtdIns4P-positive membrane to recruit NLRP3 in a manner independent of K^+^-efflux. Instead, CDC-driven K^+^ efflux is required for the subsequent recruitment of the downstream adaptor ASC. Disruption of the endoplasmic reticulum–endosome membrane contact sites (EECS) and PtdIns4P exchange between endosomes and the TGN also facilitate the NLRP3 inflammasome activation. In contrast, imiquimod promotes NLRP3 recruitment to the remodeled TGN in a K^+^ efflux-independent manner. ASC undergoes polymerization and recruits the protease caspase-1. Caspase-1 self-cleaves into p20 and p10 fragment to assemble into the mature caspase-1, which activates the proinflammatory cytokines including interleukin (IL)-1β and the pore-forming protein gasdermin D (GSDMD) to induce pyroptosis.

Inflammasomes are multiprotein complexes that drive inflammatory responses in response to infection and cellular stress^1^. NLR family pyrin domain containing 3 (NLRP3, also known as Cryopyrin, NALP3, CIAS1, and PYPAF1) is one of the most well characterized inflammasome sensors^2^. NLRP3 activation usually requires two signals^3^. Signal 1, also known as a priming signal, triggers the NF-κB signaling and induces the transcription of key components including NLRP3 and proinflammatory cytokines^4,5^. Signal 2, also known as the activation signal or stimulus, are a panel of pathogen-associated molecular patterns (PAMPs) and damage-associated molecular patterns (DAMPs) that promote NLRP3 oligomerization and activation^6^. Activated NLRP3 recruits the adaptor protein apoptosis-associated speck-like protein containing a CARD (ASC, also known as PYCARD)^7^. ASC undergoes prion-like polymerization and recruits the protease caspase-1^8,9^. Caspase-1 self-cleaves into p20 and p10 fragments, which assemble into the mature caspase-1. Mature caspase-1 mediates the proteolytic activation of proinflammatory cytokines including interleukin (IL)-1β and IL-18, and the pore-forming protein gasdermin D (GSDMD) to trigger pyroptosis, an inflammatory form of cell death^10^ (Fig. 1). Among inflammasome sensors, NLRP3 is distinguished by its ability to respond to a broad spectrum of stimuli with substantial chemical and structural diversity. As a result, the NLRP3 inflammasome plays crucial roles in a wide range of infectious and inflammatory diseases including cancers, neurodegenerative disorders, metabolic imbalance, and autoinflammatory diseases^11^. In this review, we highlight recent advances on the identification and mechanistic characterization of diverse groups of NLRP3 stimuli and discuss the technical challenges and considerations in defining new NLRP3 stimuli (Box 1). Representative classes of experimentally verified NLRP3 stimuli are summarized in Table 1. For a comprehensive overview of the underlying mechanisms and their implications in human disease, readers are referred to recent reviews^12–15^.

**Table 1:** Representative classes of NLRP3 stimuli

Name	Structure	Source	NLRP3 inflammasome stimulation			
			Cell type	Assay	Proposed mechanism	References
**Small molecules**						
Nigericin		*Streptomyces hygroscopicus*	Murine BMDMs	Caspase-1 cleavage IL-1β cleavage IL-1β secretion	K^+^ efflux TGN remodeling Endosome remodeling Mitochondrial DNA release	^16,18–22,24^ ChemSpider 10196461
ATP		Self	Murine BMDMs	Caspase-1 cleavage IL-1β cleavage IL-1β secretion	K^+^ efflux TGN remodeling Mitochondrial DNA release	^16,18–20,24^ ChemSpider 5742
Imiquimod		Synthetic	Murine BMDCs	Caspase-1 cleavage IL-1β cleavage IL-1β secretion	K^+^ efflux-independent ROS production TGN remodeling	^20,31,32^ ChemSpider 51809
CL097		Synthetic	Murine BMDCs	Caspase-1 cleavage IL-1β cleavage IL-1β secretion	K^+^ efflux-independent ROS production TGN remodeling	^20,32^ ChemSpider 9754384
**Particulate matters**						
Monosodium urate (MSU)		Self	Primary human monocytes THP-1 Murine peritoneal macrophages	Caspase-1 cleavage IL-1β cleavage IL-18 cleavage IL-1β secretion	Phagocytosis Lysosomal destabilization Lysosomal cathepsin B release	^35^ ChemSpider 8326
Calcium pyrophosphate dihydrate		Self	Primary human monocytes THP-1 Murine peritoneal macrophages	Caspase-1 cleavage IL-1β cleavage IL-18 cleavage IL-1β secretion	Phagocytosis Lysosomal destabilization Lysosomal cathepsin B release	^35^ ChemSpider 14690612
Silica	SiO2	Environment	Human PBMCs Murine BMDMs	Caspase-1 cleavage IL-1β cleavage IL-1β secretion	Phagocytosis Lysosomal destabilization Lysosomal cathepsin B release	^36^
Aluminum salts	Al(OH)_3_	Environment	Human PBMCs Murine BMDMs	IL-1β cleavage IL-1β secretion	Phagocytosis Lysosomal destabilization Lysosomal cathepsin B release	^36^
Cholesterol crystals		Self	Human PBMCs	Caspase-1 cleavage IL-1β cleavage IL-1β secretion	Phagocytosis Lysosomal destabilization Lysosomal cathepsin B and cathepsin L release	^125^ ChemSpider 5775
**Peptides**						
Amyloid-β		Self	Primary mouse microglia Immortalized microglia cell line Murine BMDMs	Caspase-1 cleavage IL-1β secretion	Lysosomal cathepsin B release	^48^ ChemSpider 17292072 (Amyloid β-Peptide, 1-40)
Gramicidin		*Brevibacillus brevis*	Primary human monocytes Murine BMDMs	Caspase-1 cleavage IL-1β secretion	K^+^ efflux TGN remodeling	^20,19,52^ ChemSpider 17288928 (Gramicidin A)
Muramyl dipeptide (MDP)		Various bacteria	Primary human macrophages 293 T NLRP3 + ASC + CASP1 + IL-1β	IL-1β cleavage IL-1β secretion	/	^31^*,^55^,^56^* ChemSpider 9794910
**Proteins**						
Haemolysin BL (HBL)		*Bacillus cereus*	Murine BMDMs	Caspase-1 cleavage IL-1β secretion IL-18 secretion	K^+^ efflux	^59^ pdb_00007nmq (monomer)
Non-hemolytic enterotoxin (NHE)	N/A	*Bacillus cereus*	Murine BMDMs	Caspase-1 cleavage GSDMD cleavage IL-1β secretion IL-18 secretion	K^+^ efflux	^60^
Perfringolysin O (PFO)		*Clostridium perfringens*	Murine BMDMs 293 T NLRP3 + ASC + CASP1	Caspase-1 cleavage IL-1β cleavage IL-1β secretion	K^+^ efflux TGN remodeling	^61,68,74^ pdb_00001pfo (homodimer)
Anthrolysin O (ALO)	pdb_00003cqf (monomer)	*Bacillus anthracis*	293 T NLRP3 + ASC + CASP1	Caspase-1 cleavage	K^+^ efflux TGN remodeling	^68^
Streptolysin O (SLO)	pdb_00004hsc (monomer)	*Streptococcus pyogenes*	Murine BMDMs 293 T NLRP3 + ASC + CASP1	Caspase-1 cleavage IL-1β secretion	K^+^ efflux TGN remodeling	^68,76^
Pneumolysin (PLY)	pdb_00005aod (monomer)	*Streptococcus pneumoniae*	Murine BMDMs Murine bone marrow neutrophils 293 T NLRP3 + ASC + CASP1	Caspase-1 cleavage IL-1β secretion	K^+^ efflux TGN remodeling	^56,68,75^
Listeriolysin O (LLO)	pdb_00004cdb (monomer)	*Listeria monocytogenes*	Human PBMCs Murine BMDMs	Caspase-1 cleavage IL-1β cleavage IL-1β secretion	K^+^ efflux	^70,71,126^*,^127^*
α-hemolysin (Hla)		*Staphylococcus aureus*	THP-1 Murine peritoneal macrophages Murine lung cells	Caspase-1 cleavage IL-1β cleavage IL-1β secretion	K^+^ efflux	^85,128^ pdb_00007ahl (homoheptamer)
α-toxin	N/A	*Clostridium septicum*	Murine BMDMs	Caspase-1 cleavage GSDMD cleavage IL-1β secretion	K^+^ efflux Mg^2+^ efflux	^87^
Aerolysin		*Aeromonas hydrophila*	Murine BMDMs	Caspase-1 cleavage IL-1β cleavage IL-1β secretion	K^+^ efflux	^129^ pdb_00005jzw (homotetradecamer)
Thermostable direct hemolysins (TDHs)	N/A	*Vibrio parahaemolyticus*	Murine BMDMs	Caspase-1 cleavage IL-1β cleavage IL-1β secretion	/	^91^
Toxin B (TcdB)		*Clostridioides difficile*	Human MDMs BLaER1 THP-1	Caspase-1 cleavage IL-1β cleavage IL-1β secretion	/	^95^ pdb_00007lou (monomer)
Phospholipase C (PLC)		*Clostridium perfringens*	Murine BMDMs	Caspase-1 cleavage GSDMD cleavage IL-1β secretion	K^+^ efflux Lysosomal destabilization	^61^ pdb_00001ca1 (monomer)
M2		Influenza A virus	Murine BMDMs Murine BMDCs	Caspase-1 cleavage IL-1β cleavage IL-1β secretion	Ion perturbation at the Golgi	^102^ pdb_00003bkd (transmembrane domain, homotetramer)
ORF3a (SARS-CoV)		SARS-CoV	THP-1 Murine BMDMs	IL-1β cleavage IL-1β secretion	K^+^ efflux ROS production TRAF3-dependent ubiquitination of ASC	^103^*,^104^,^105^ pdb_00008eqs (heterotetramer)
ORF3a (SARS-CoV-2)		SARS-CoV-2	HEK293 T (?) A549 (?)	Caspase-1 cleavage IL-1β cleavage IL-1β secretion	K^+^ efflux	^103^*,^106^ pdb_00006xdc (homodimer)
Membrane attack complex		Self	Murine BMDCs Primary human lung epithelial cells Human MDMs THP-1	Caspase-1 cleavage GSDMD cleavage IL-1β cleavage IL-1β secretion	K^+^ efflux Ca^2+^ influx TGN remodeling	^109–111^ pdb_00006h03 (heterotetracosamer)
**Nuclei acids and related products**						
Bacterial and viral DNA	/	Various bacteria and viruses	Human primary monocytes BLaER1 Monocytes	Caspase-1 cleavage IL-1β cleavage IL-1β secretion	cGAS-STING-mediated lysosomal cell death K^+^ efflux	^113^
Bacterial RNA	/	Various bacteria	Murine peritoneal macrophages Murine BMDMs	Caspase-1 cleavage IL-1β secretion	K^+^ efflux Phagocytosis of bacteria Lysosomal acidification	^31,116,117,130^*
Bacterial DNA: RNA hybrid	/	Various bacteria	Murine BMDMs Murine BMDCs	Caspase-1 cleavage IL-1β cleavage IL-1β secretion	K^+^ efflux Phagocytosis of bacteria Lysosomal acidification	^116^
Cyclic-di-GMP		Various bacteria	THP-1 Murine BMDMs	Caspase-1 cleavage IL-1β cleavage IL-1β secretion	K^+^ efflux ROS-independent	^131^ ChemSpider 4883312
Cyclic-di-AMP		Various bacteria	THP-1 Murine BMDMs	Caspase-1 cleavage IL-1β cleavage IL-1β secretion	K^+^ efflux ROS-independent	^131^ ChemSpider 9333199
poly(I:C)	/	Synthetic	Murine BMDMs	IL-1β secretion	Endosomal acidification	^130^*,^132^

*References marked with an asterisk present conflicting evidence suggesting that the indicated stimuli did not activate the NLRP3 inflammasome.

*NA* not available. *TGN* *trans*-Golgi network. *BMDMs* bone marrow-derived macrophages. *BMDCs* bone marrow-derived dendritic cells. *PBMCs* peripheral blood mononuclear cells. *MDMs* monocyte-derived macrophages. *CASP1* caspase-1. *ROS* reactive oxygen species.

Box 1 Defining new NLRP3 inflammasome stimuli Due to the lack of direct binding between NLRP3 and most of its stimuli, it is conceptually more challenging to define bona fide stimuli for NLRP3 compared to other sensors. Standardized assays, along with careful consideration of factors that may interfere with results, are therefore crucial for studying NLRP3 stimuli.

Assays and readouts. The activation of the NLRP3 inflammasome is a complex process involving the priming step and the sequential activation of the sensor (NLRP3), adaptor (ASC), protease (caspase-1), cytokines (e.g., IL-β and IL-18), and others (e.g., GSDMD). Specific assays have been established to examine the activation of each of these steps. The activation of NLRP3 can be examined by its transition from the diffusing cytosolic state to the aggregated state via imaging^20^. The activation of ASC can be assessed with the formation of a single ‘speck’ per cell via imaging^8,9^ or with its conversion from monomeric to high-molecular-weight oligomeric form with crosslinking and immunoblotting^118,133,134^. The activation of caspase-1, IL-1β, IL-18, and GSDMD can be detected via their proteolytic cleavage with immunoblotting, typically in cell culture supernatants to detect the secreted forms^135^.

Considerations and pitfalls. Another commonly used assay is the measurement of secreted IL-1β in the supernatants by enzyme-linked immunosorbent assay (ELISA). Because of its quantitative nature, IL-1β ELISA is ideal for characterizing partial phenotypes. However, IL-1β antibodies cannot distinguish between the full-length and cleaved forms of IL-1β^135^, both of which are released into the supernatant^16^. Similarly, cell viability assays, such as LDH release assay, cannot distinguish pyroptosis from other forms of cell death^136,137^. Therefore, it is recommended to perform caspase-1 or IL-1β immunoblotting in conjunction with these assays to fully verify inflammasome activation. When using pharmacological inhibitors or genetic manipulations, it is also crucial to determine whether observed defects affect the priming step rather than the activation step. This can be verified by monitoring pro-IL-1β induction via cell extract immunoblotting or by using reconstituted cell systems in which the priming step is bypassed through NLRP3 overexpression^4,138^.

Cell types and NLRP3 dependency. NLRP3 is highly expressed in cells of the myeloid lineage. Consequently, primary or immortalized bone marrow-derived macrophages (BMDMs), primary bone marrow-derived dendritic cells (BMDCs), and the human THP-1 cell line are among the most commonly used models. Recent studies, however, have revealed that the NLRP3 inflammasome is also expressed in a variety of other tissues and cell types^14^, and that its activation mechanisms can vary depending on the species and cell types involved^95,139–141^. The development of new cell and animal models is thereby needed to identify new stimuli and mechanisms. Finally, when defining novel NLRP3 stimuli, an important but often overlooked control is to verify whether the observed activity is indirectly mediated by cellular stress through the ATP–P2X_7_–NLRP3 axis. This can be achieved by either examining primary *P2X*_*7*_^-/-^ BMDMs/BMDCs^142^ or cell lines lacking a functional P2X_7_ signaling pathway (e.g., THP-1^135^ or HEK293T/HeLa reconstituted with NLRP3, ASC, and caspase-1^20^).

## Small-molecule stimuli

**Fig. 2:**
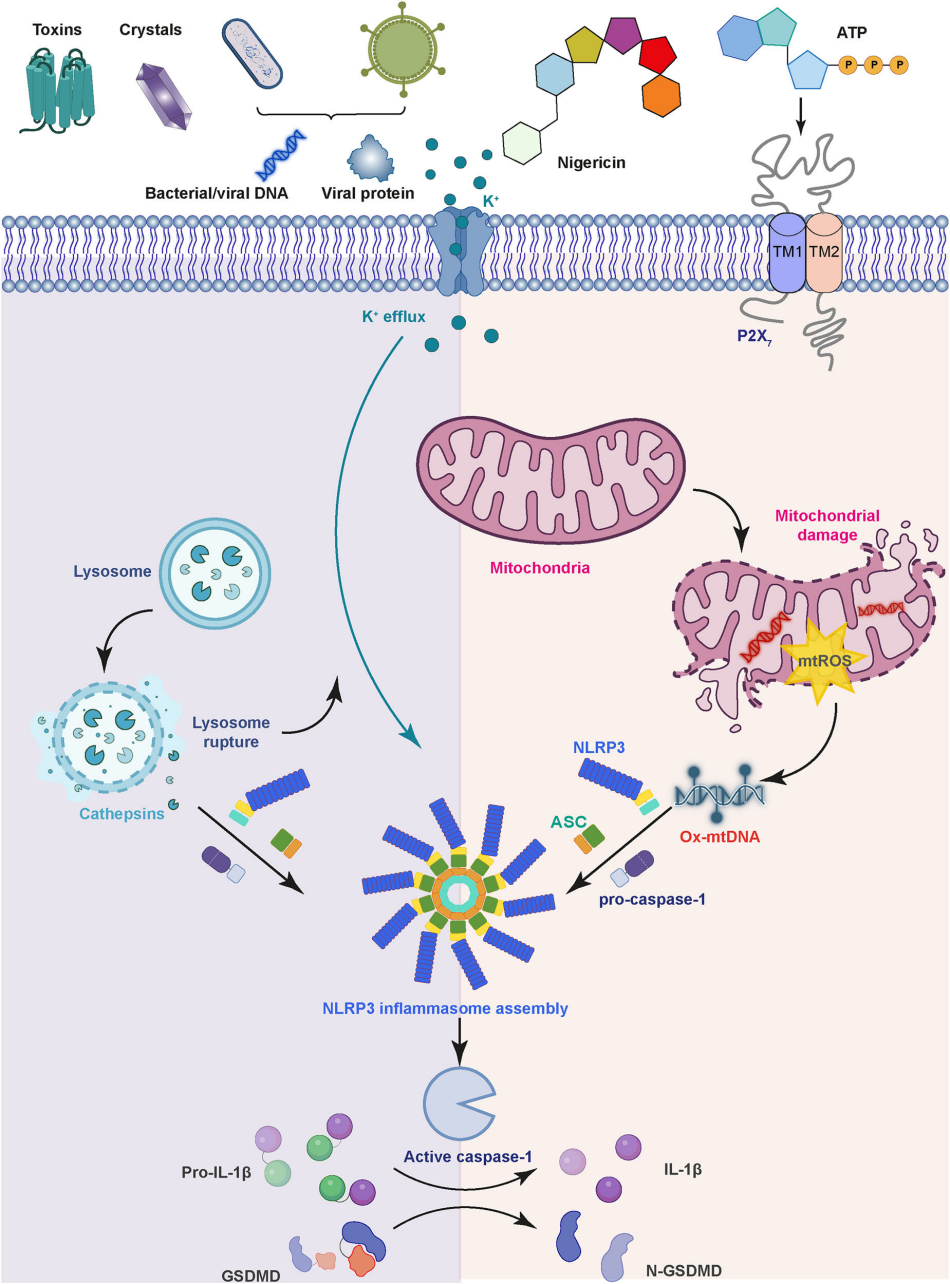
Model of lysosomal and mitochondrial disruption by NLRP3 stimuli. Various bacterial toxins, crystals, bacterial/viral DNA, and other types of NLRP3 stimuli can be internalized through phagocytosis. This results in lysosomal rupture that releases its contents including cathepsin proteases to mediate NLRP3 activation in the presence of K^+^ efflux. Other stimuli including nigericin and ATP induce mitochondrial damage that releases oxidized mitochondrial DNA to initiate the NLRP3 inflammasome activation.

The small-molecule stimuli nigericin and adenosine triphosphate (ATP) are among the earliest characterized stimuli^16^ and serve as the gold-standard NLRP3 stimuli in many studies due to their commercial availability and high potency. Nigericin is a polyether antibiotic derived from the Gram-positive soil bacterium *Streptomyces hygroscopicus*^17^. It acts as a potassium (K^+^)/ hydrogen (H^+^) ionophore and drives K^+^ efflux^18^, an essential cellular process for NLRP3 inflammasome activation by nigericin and many other stimuli^19^. Several other polyether ionophores, including A204 from *Streptomyces albus* and lasalocid from *Streptomyces lasaliensis*, also triggered K^+^ efflux and induced IL-1β secretion in macrophages^18^, although it has not been confirmed whether these activities are NLRP3-dependent. In contrast, monensin, an ionophore with similar structure with nigericin but caused sodium (Na^+^) influx instead of K^+^ efflux, failed to activate the NLRP3 inflammasome^19^. The ability of nigericin to disrupt organelle homeostasis also plays a key role in NLRP3 activation. Nigericin stimulation triggered the dispersion of the *trans*-Golgi network (TGN) into multiple vesicles that recruited NLRP3 through an ionic bonding between TGN-localized phospholipid PtdIns4P and a polybasic region in NLRP3, which was essential for the assembly of the inflammasome complex^20^. K^+^ efflux was dispensable for nigericin-mediated TGN remodeling, but was required for the remodeled TGN to recruit NLRP3^20^, possibly by lowering intracellular ionic strength to promote the ionic bonding. Thus, nigericin triggers two parallel events: K^+^ efflux and TGN remodeling, both of which are essential for its NLRP3-stimulating activity (Fig. 1). Recent studies demonstrate that nigericin and other NLRP3 stimuli also disrupted the endoplasmic reticulum–endosome membrane contact sites (EECS)^21,22^ (Fig. 1) and triggered the production of reactive oxygen species (ROS) that oxidized mitochondrial DNA through mitochondrial damage^23,24^ (Fig. 2). Phase separation mediated by palmitoyl transferase ZDHHC7-driven NLRP3 palmitoylation has also been implicated in nigericin-induced NLRP3 activation^25^. The exact molecular mechanisms governing nigericin-mediated organelle remodeling and damage, however, remain mysterious. Therefore, further investigation is required to clarify how different organelles contribute to NLRP3 inflammasome activation.

ATP is the primary intracellular energy currency sustaining metabolic homeostasis^26^. However, cellular stress, tissue injury^27^, and inflammatory conditions^28^ release it into the extracellular compartment via channel pores or lysosomal exocytosis, where it functions as a DAMP^27^. Extracellular ATP engages the purinergic receptor P2X_7_ on the plasma membrane to trigger K^+^ efflux, Na^+^ influx, and calcium (Ca^2+^) influx^29^, with K^+^ efflux being the critical factor in activating NLRP3^19^. P2X_7_ also forms a pore-like structure that permits the passage of hydrophilic molecules up to ~900 Da^29,30^. This may enhance ATP release to establish a positive feedback loop^28^. Similar to nigericin, ATP stimulation resulted in the disassembly of the TGN into multiple giant vesicles, which serve as platform to recruit NLRP3^20^. The presence of P2X_7_ is essential for TGN remodeling driven by ATP but dispensable for TGN remodeling driven by nigericin^20^, suggesting that these two stimuli may utilize distinct mechanisms to reorganize this signaling-hub organelle.

While K^+^ efflux is essential for the majority of known NLRP3 stimuli^20^, a small group of stimuli, represented by imiquimod (also known as R837)^31^ and CL097, activated the NLRP3 inflammasome in a K^+^ efflux-independent manner^32^, an activity independent of their previously known roles as Toll-like receptor 7 (TLR7) agonists^32,33^. Imiquimod drove cytosolic and mitochondrial reactive oxygen species (ROS) production by inhibiting the activity of NRH:quinone oxidoreductase 2 (NQO2) and mitochondrial respiratory complex I, respectively^32^, leading to ROS-mediated protein oxidization^32^. Combined treatment with NQO2 and complex I inhibitors, however, failed to activate the NLRP3 pathway, indicating that imiquimod also operates through other mechanisms^32^. Similar to nigericin and ATP, imiquimod and CL097 induced potent TGN dispersion and the subsequent recruitment of NLRP3^20^. Disruption of NLRP3 recruitment to the dispersed TGN abolished imiquimod-induced NLRP3 inflammasome activation^20^, suggesting that TGN remodeling also plays a key role in NLRP3 activation by K^+^ efflux-independent stimuli. Notably, imiquimod and CL097 drove a much more potent TGN dispersion than nigericin or ATP^20^, which may explain why these stimuli facilitated the recruitment of NLRP3 without K^+^ efflux. A recent study identified the bacteria-derived short-chain fatty acid butyrate as another K^+^ efflux-independent stimulus^34^. Butyrate inhibited the histone deacetylases (HDACs) to induce caspase-8-dependent NLRP3 inflammasome activation^34^, highlighting that K^+^ efflux-independent stimuli may utilize diverse molecular mechanisms to activate the NLRP3 sensor.

## Particulate matter stimuli

This class contains a number of insoluble crystals and particles such as monosodium urate (MSU)^35^, calcium pyrophosphate crystals^35^, and silica^36^. Released by stressed or dying cells, the decomposition of these particulate matters in various organs result in severe chronic inflammatory disorders^35^. For example, cholesterol crystals drive NLRP3-dependent inflammatory cytokine production and facilitate cardiovascular pathology^37,38^. One of the most characterized stimuli in this group is MSU. In Injured cells, purines liberated from degraded DNA and RNA are converted to uric acid, the protonated form of MSU^39^. Accumulation of uric acid in patients with hyperuricemia (characterized by elevated blood urate levels) prompts the formation of MSU crystals^39^, whose precipitation in joints cause gout, a chronic form of inflammatory arthritis^40^. MSU and other particulate matter stimuli were internalized by monocytes and macrophages through phagocytosis, leading to lysosomal destabilization and leakage of lysosomal contents^36^. Early studies have suggested that the release of the lysosomal cysteine protease cathepsin B was essential for NLRP3 activation based on the effects of cathepsin B inhibitor CA-074Me^36,41,42^ (Fig. 2). However, the specificity of CA-074Me has been questioned in later studies, and whether other cathepsins play redundant roles with cathepsin B still need to be fully characterized^43,44^. Nevertheless, the central role of the NLRP3 inflammasome in gout development was supported by the effectiveness of IL-1 inhibitors in managing gout flares^40^.

## Peptide stimuli

Peptide-derived NLRP3 stimuli encompass both endogenous and exogenous peptides. Amyloid-β is a self-derived peptide associated with the Alzheimer’s disease^45,46^, although its exact role in disease progression remains controversial^47^. In microglia, amyloid-β was internalized via phagocytosis and trafficked to the lysosomes^48^. Similar to particulate matters, amyloid-β caused lysosomal destabilization to activate NLRP3^48^. Amyloid-β also induced ROS production that augments NLRP3 activation^49^. Another endogenous peptide stimulus is LL-37, a 37-residue cationic antimicrobial peptide generated from the C-terminal domain of human CAP18 precursor^50^. LL-37 was endocytosed through the P2X_7_ channel and transported to lysosomes, where it drove lysosomal dysfunction and subsequent NLRP3 activation in a K^+^ efflux-dependent manner^51^. Exogenous peptide stimuli are represented by gramicidins from the Gram-positive soil bacterium *Brevibacillus brevis*^52^. Gramicidins are a group of non-ribosomal 15-residue peptides consisting of alternating L- and D-amino acids^53^. Commercially available gramicidins consist mainly of gramicidin A with a small amount of other gramicidins that differ at residue 11^53^. Gramicidins form membrane-spanning cation-selective channels^54^ and induce K^+^ efflux to support NLRP3 inflammasome activation^19^. Similar to nigericin, gramicidins also induce TGN remodeling^20^, although whether non-ribosomal peptide ionophores and small-molecule ionophores mediate organelle reorganization through similar or different mechanisms remains unknown. Muramyl dipeptide (MDP), a component of bacterial peptidoglycan, has been proposed as another bacteria-derived peptide stimulus^55^. However, its role remains debated since recent studies have suggested that MDP may function in the priming step instead of the activation step^31,56^.

## Protein stimuli

A wide range of bacterial toxins activate the NLRP3 inflammasome. Bacterial toxin stimuli may represent the most effective tools for studying NLRP3 inflammasome activation mechanisms due to their protein nature, which allows ease of genetic manipulation and access to established biochemical and cell biological tools. Pore-forming toxins (PFTs) are a major group of bacterial toxins and they act as virulence factors secreted by pathogenic bacteria before forming pores on the host cells^57^. PFTs are grouped into two classes, α-PFTs and β-PFTs, based on whether the membrane-spanning regions are composed of α-helices or β-barrels, respectively^58^. NLRP3 inflammasome stimuli have been found in both classes. The tripartite enterotoxin hemolysin BL (HBL), an α-PFT produced by the Gram-positive bacterium *Bacillus cereus*, activated the NLRP3 inflammasome during bacterial infection and as a recombinant protein^59^. The sequential assembly of its three subunits was essential for HBL to form pores on the host cell surface to promote K^+^ efflux and drive NLRP3 activation^59^. Another tripartite α-PFT secreted by *B. cereus*, non-hemolytic enterotoxin (NHE), activated the NLRP3 inflammasome in a manner similar to HBL^60^. Interestingly, while recombinant NHE activated the NLRP3 inflammasome within 3 hours, overnight *B. cereus* infection was required for endogenous NHE to activate NLRP3^60^. The significantly slower kinetics of endogenous NHE as an NLRP3 stimulus was not due to a delay in expression or secretion, as NHE was constitutively secreted by *B. cereus*^59^. A similar case was observed with a non-PFT toxin phospholipase C (PLC, also known as lecithinase) from *Clostridium perfringens*, which required overnight bacterial infection to activate the NLRP3 inflammasome^61^. Genetic ablation of NLRP3 or caspase-1 extended the survival time of mice injected with PLC^61^, suggesting that PLC-mediated NLRP3 activation contributes to the disease progression. How bacteria regulate the timing of different virulence factors to activate NLRP3 remains unexplored. However, these findings suggest that a single bacterial species may encode multiple NLRP3 stimuli to drive inflammasome activation at different stages of infection, which may facilitate bacterial dissemination within the host.

Cholesterol-dependent cytolysins (CDCs) constitute the largest family of PFTs and belong to the β-PFT class^58^. CDCs are crucial virulence factors encoded by a large variety of Gram-positive bacteria that cause diseases including anthrax, gas gangrene, pharyngitis, pneumonia, meningitis, and sepsis^62–67^. A number of CDCs, represented by perfringolysin O (PFO) from *C. perfringens*, activated the NLRP3 inflammasome^56,61,68–77^. These CDCs bound to cholesterol on the host cell plasma membrane to form pores^78^ and induced ion fluxes including K^+^ efflux and Ca^2+^ influx^79,80^. CDC toxins were then internalized inside host cells and trafficked to the TGN, where they peeled away PtdIns4P-negative TGN membrane into vesicles, thus exposing the remaining PtdIns4P-positive TGN membrane to recruit NLRP3^68^ (Fig. 1). In contrast, desulfolysin (DLY), a CDC secreted by the free-living Gram-negative bacterium *Desulfobulbus propionicus*^81^, did not traffic to the TGN and as a result failed to activate NLRP3 signaling^68^. DLY possessed potent pore-forming activity to drive K^+^ efflux^68^, indicating that K^+^ efflux is essential but insufficient to support NLRP3 inflammasome activation. Consistently, while K^+^ efflux was required for pathogenic bacteria-derived CDCs to activate the NLRP3 inflammasome^79^, it was only required for the recruitment of ASC but not for TGN remodeling or NLRP3 recruitment^68^. This is in marked contrast to the requirement of K^+^ efflux in mediating NLRP3 recruitment following nigericin stimulation^20^, which may be caused by different TGN remodeling mechanisms.

Several other β-PFTs serve as NLRP3 stimuli. α-hemolysin (Hla) is secreted by the Gram-positive *Staphylococcus aureus* that causes pneumonia, skin infection, and toxic shock syndrome^82,83^. Hla binds to host cells in both metalloprotease ADAM10-dependent (at low concentration) and -independent (at high concentration) manner^84^. Recombinant Hla, but not its inactive mutant H35L, induced NLRP3-dependent caspase-1 cleavage and IL-1β secretion in the human monocyte-derived cell line THP-1 via K^+^ efflux^85^. Hla treatment resulted in rapid release of cellular ATP^86^, thus raising the question of whether Hla activates NLRP3 indirectly through the ATP–P2X_7_–NLRP3 axis. Another β-PFT, α-toxin secreted by Gram-positive *Clostridium septicum*, triggered NLRP3-dependent caspase-1 and GSDMD cleavage in both human and murine macrophages^87^. Unlike CDCs, the cytosolic entry of α-toxin was not required for this activity^87^. Instead, its interaction with glycosylphosphatidylinositol (GPI)-anchored proteins on the host plasma membrane^88^ and the resulting K^+^ efflux were required for the inflammasome stimulation^87^. Interestingly, both α-toxin and PFO also induced magnesium (Mg^2+^) efflux, an effect not detected for ATP treatment^87^. Blocking either K^+^ efflux or Mg^2+^ efflux repressed α-toxin-mediated inflammasome activation^87^. Further studies are required to investigate why α-toxin requires an additional form of ion flux to drive NLRP3 inflammasome activation. While α-toxin has not been found in any other clostridial species, it is structurally similar to aerolysin, a β-PFT from Gram-negative *Aeromonas hydrophila*^89^. Aerolysin also activated the NLRP3 inflammasome via K^+^ efflux^19,90^, although whether it employs mechanisms similar to those of α-toxin has yet to be experimentally verified.

Besides PFTs, other classes of bacterial toxins have been reported to activate NLRP3. Infection of the Gram-negative bacterium *V. parahaemolyticus* induced potent NLRP3 inflammasome activation, an activity significantly attenuated when its encoded toxins thermostable direct hemolysin A (TdhA) and S (TdhS) were genetically ablated^91^, suggesting that these two toxins function redundantly to drive NLRP3 activation. Stimulation with recombinant TdhA and TdhS will be necessary to determine whether these toxins can directly activate NLRP3 in the absence of other bacterial factors. *Clostridioides difficile* toxin A (TcdA) and B (TcdB) are key virulence factors that promote pseudomembranous colitis after *C. difficile* infection^92^. TcdA and TcdB enter cells via receptor-mediated endocytosis and inactivate small Rho GTPases through glucosylation, ultimately leading to cell death^93^. While TcdB activated the pyrin inflammasome through RhoA glucosylation in murine macrophages^94^, it activated the NLRP3 inflammasome in human monocyte-derived macrophages (hMDM) instead^95^. Surprisingly, the glucosyltransferase activity of TcdB was not required for the latter activity^95^, indicating that it activated NLRP3 with a mechanism distinct from its pyrin-stimulating activity. In contrast, TcdA did not trigger NLRP3 activation for unknown reasons despite its high similarity with TcdB^95^. Previously, TcdB but not TcdA was found to be essential for *C. difficile* virulence in a hamster disease model^96^. It will be interesting to investigate whether the distinct capacities of these two toxins to activate the NLRP3 inflammasome influence their differential contributions to *C. difficile*-driven pathogenesis.

In addition to bacterial toxins, a large group of bacterial virulence factors have been identified as NLRP3 stimuli. Examples include cyclase toxin (CyaA) from *Bordetella pertussis*^97^, leukotoxin (LtxA) from *Aggregatibacter actinomycetemcomitans*, cytotoxic necrotizing factor 1 (CNF1) from *Escherichia coli*^98^, early secreted target with a molecular weight of 12 kDa (EST12) from *Mycobacterium tuberculosis*^99^, and the surface protein M1 from group A *Streptococcus*^100^. Whether these protein stimuli are localized to intracellular organelles to trigger NLRP3 activation or act through indirect mechanisms remains to be determined.

Several viral proteins have been reported to activate NLRP3. The first group consists of viroporins, which are diverse small ion channel proteins in viruses^101^. While influenza A virus-encoded M2 protein was located on the TGN^102^, it did not induce detectable TGN remodeling^103^. Instead, it used its proton-specific ion channel activity to export H^+^ from the acidic TGN lumen into the cytosol, thus prompting K^+^ efflux to drive caspase-1 activation and IL-1β secretion via NLRP3^102^. Another viroporin, open reading frame 3a (ORF3a) encoded by SARS-CoV, was reported to activate the NLRP3 inflammasome, although whether its ion channel activity is required for this activity is debated^104,105^. The homologue of ORF3a in SARS-CoV-2 was also proposed to serve as an NLRP3 stimulus^106^. However, this study used the non-myeloid cell lines HEK293T and A549 without reconstitution of inflammasome components^106^. Thus, the conclusion needs to be further validated using primary or immortalized myeloid cells with the NLRP3 inflammasome pathway. In contrast, a recent study failed to detect NLRP3 inflammasome activation with ORF3a from either SARS-CoV or SARS-CoV-2, consistent with their inability to remodel the PtdIns4P-positive TGN membrane^103^. Future studies using WT and Δ*orf3a* virus will help determine whether ORF3a activate the NLRP3 inflammasome during infection. Other viral proteins reported to activate the NLRP3 inflammasome include non-structural protein 6 (NSP6) of SARS-CoV-2, which targets the vacuolar ATPase ATP6AP1 to impair lysosomal acidification^107^, and nucleocapsid (N) protein of SARS-CoV-2, which directly interacted with NLRP3 via the 260–340-residue region in N^108^.

Self-derived proteins also act as NLRP3 stimuli. The membrane attack complex (MAC) in the complement system activated the NLRP3 inflammasome^109,110^ after being internalized inside cells and inducing TGN dispersion^111^. MAC is structurally related to bacterial CDC toxins^58^, thus raising the question of whether MAC may use a similar peeling membrane mechanism to remodel the TGN. The ability of MAC to activate the NLRP3 inflammasome suggests that this proinflammatory response may contribute to the acute and chronic inflammatory disorders including rheumatoid arthritis^111^. Another self-derived protein NLRP3 agonist is β-2-microglobulin (β2m), whose phagocytosis and accumulation in the lysosomes activated the NLRP3 inflammasome, thereby promoting the progression of multiple myeloma^112^.

## Nucleic acid and related molecular stimuli

Both DNA and RNA have been reported to activate the NLRP3 inflammasome either directly or indirectly through other signaling pathways. Although cytosolic DNA primarily activates the AIM2 inflammasome, in human monocytes from peripheral blood and BLaER1 monocytes, cytosolic DNA derived from the Gram-negative bacterium *Francisella novicida* and vaccinia virus activated NLRP3 instead of AIM2, by triggering lysosomal cell death and K^+^ efflux through the cGAS-STING pathway^113^. Self-double-stranded DNA (dsDNA) has also been reported to activate NLRP3 in conjunction with anti-dsDNA antibodies, promoting autoimmune disorders including systemic lupus erythematosus^114^. Bacterial RNA extracted from both Gram-positive and Gram-negative bacteria^31^, including mRNA, rRNA, and tRNA^115^, also activated the NLRP3 inflammasome. Mechanistically, bacteria were phagocytosed, and bacterial RNA and DNA:RNA hybrids were released from lysosomes into the cytosol, where they were recruited to NLRP3 and mediate its activation^116^. In contrast, RNA extracted from mouse liver failed to activate the NLRP3 inflammasome^31^. How mammalian cells distinguish pathogenic RNA from self RNA has not been fully established, although 3’ polyadenylation of RNA and differences in nucleoside modifications were proposed as potential mechanisns^31,117^. Bacterial mRNA was also shown to potentiate the noncanonical activation of NLRP3 downstream of caspase-11-mediated sensing of intracellular lipopolysaccharide (LPS)^118^.

## Conclusion and perspectives

Studies over the past two decades have revealed an explosion in the number and diversity of molecules that activate the NLRP3 inflammasome, providing new insights into the various mechanisms underlying its activation. By sensing disruptions in organelle and ion homeostasis, the NLRP3 sensor functions as a highly flexible surveillance system capable of detecting a broad spectrum of danger and invasion signals, an evolutionary advantage that allows mammals to respond to diverse pathogens and environmental stresses. Despite significant progress, several fundamental questions in this field remain unresolved.

### Physiological relevance of NLRP3 stimuli

While many NLRP3 activators, such as bacterial toxins and MSU, have well-established roles in health and disease, many others lack clear physiological significance. For instance, nigericin and imiquimod are widely used as representative K^+^ efflux-dependent and -independent stimuli, respectively, and have been invaluable tools for studying inflammasome biology. However, nigericin is produced by a free-living soil bacterium, while imiquimod is a synthetic compound. Recent studies suggest that these molecules may engage unique molecular mechanisms distinct from those of other K⁺ efflux-dependent or -independent stimuli^34,68^. An important area for future investigation is whether physiologically relevant agonists that mimic the effects of these stimuli exist during infection, tissue damage, or metabolic stress.

### Uncharacterized NLRP3 stimuli in cancer and other inflammatory diseases

While pathogen-derived NLRP3 activators have been extensively studied, the identities and mechanisms of NLRP3 stimuli driving sterile inflammatory diseases remain poorly understood. The NLRP3 inflammasome promoted the progression and severity of breast cancer, lung metastasis, fibrosarcoma, and gastric carcinoma, while inhibiting tumorigenesis in colitis-associated colorectal cancer^119^. With the exception of *Helicobacter felis*-induced gastric cancer, these malignancies lack a clear infectious component. It remains to be determined which sterile stimuli, such as those arising from oncogene activation or tumor suppressor loss, drive NLRP3 signaling in these contexts. In addition, gut microbiota alterations and intestinal injury recruited monocytes and promoted NLRP3-dependent inflammatory responses in the intestine^120,121^. Consistent with this observation, NLRP3 deficiency partially rescued gut microbiota dysbiosis-induced intestinal injury and inflammation^120,121^. However, the nature of microbiota-derived NLRP3 stimuli remains poorly understood. Activation of the NLRP3 inflammasome also promoted autoimmune diseases such as IgA nephropathy induced by abnormal IgA1 accumulation, although the exact direct stimuli and mechanisms are unclear^122–124^. Finally, although NLRP3 contributes to metabolic disorders and neurodegenerative diseases^11^, the identity of the endogenous stimuli responsible for its activation in these conditions remains elusive. A deeper understanding of the underlying molecular and cellular mechanisms will be essential for identifying therapeutic targets to address these major human health challenge.
